# Restriction modification systems as engines of diversity

**DOI:** 10.3389/fmicb.2015.00528

**Published:** 2015-06-02

**Authors:** Kim Sneppen, Szabolcs Semsey, Aswin S. N. Seshasayee, Sandeep Krishna

**Affiliations:** ^1^Center for Models of Life, Niels Bohr InstituteCopenhagen, Denmark; ^2^National Centre for Biological SciencesBangalore, India; ^3^Simons Centre for the Study of Living Machines, National Centre for Biological SciencesBangalore, India

**Keywords:** bacteriophage defense, epigenetic labeling, phage-bacteria interaction, ecosystem diversity, mathematical modeling, population dynamics

## Abstract

Restriction modification (RM) systems provide protection against a broad spectrum of phages. However, the likelihood of a phage permanently bypassing this can be as high as 0.1 per infection (Korona et al., 1993) which makes for a relatively weak defense. Here we argue that, apart from providing such transient defenses, RM systems can facilitate long-term coexistence of many bacterial strains. We show that this diversity can be as large as the burst size of the phage but no larger—a curious correspondence between a number at the level of species and another number at the level of individuals. Such a highly diverse and stably coexisting ecosystem is robust to substantial variation in both bacterial growth rates and strength of their RM systems, which might be one reason why quite weak RM systems exist in the wild.

## 1. Introduction

Restriction-modification (RM) systems (Arber, 1965; Rambach and Tiollais, 1974; Kruger and Bickle, 1983; Kessler and Manta, 1990) are often discussed in connection with bacterial defense against phage predation both in bacteria (Bickle and Kruger, 1993) and archaea (Vasu and Nagaraja, 2013; Makarova et al., 2014), and are the most widespread phage defense system in bacterial genomes (Makarova et al., 2014). In addition, RM systems have evolved “moonlighting roles” in recombination, metabolism, and gene regulation (Vasu and Nagaraja, 2013). An RM system consists of two enzymatic functions, that both recognize and act on a certain DNA sequence. One of the enzymes cleaves the sequence if it is not methylated, whereas the other enzyme adds methyl groups to the sequence to protect it from cleavage. Thereby, foreign non-methylated DNA entering the cell, including DNA of invading phages, will be preferentially cleaved. This provides immunity to infection, to an extent that depends on the phage as well as on the particular RM system (Korona and Levin, 1993; Korona et al., 1993).

The immunity imposed by RM systems is not perfect. Typically, a given RM-system only recognizes a few DNA segments in a given phage, and for each of these there is a chance that it gets methylated by the bacterial modification enzyme before it is cleaved. Thereby, the phage sometimes can complete a successful infection and produce a burst of progeny phage. Furthermore, when a phage survives an infection cycle, the DNA of its progeny will be methylated and, upon infection of the same bacterial strain, will be able to bypass the RM system (Arber, 1965; Korona and Levin, 1993; Korona et al., 1993). Korona et al. (1993) suggest that this transient advantage of RM systems is useful when the host first invades an environment containing new phage, because it allows time to reach a larger population size, and thus more chance for the emergence of immune mutants and/or for the formation of a biofilm or other density-dependent mechanisms that enhance phage-bacteria coexistence (Heilmann et al., 2012). However, the fact remains that the bacteria effectively “teach” the successful phage how to bypass the defense on all the siblings of the bacteria, and it is therefore a challenge to understand the long-term advantage of this widespread class of defense systems.

Here, we explore idealized conditions where RM systems could have a long-term advantage as quantified in terms of bacterial populations under steady state growth and dilution. In agreement with more complex models (Frank, 1994; Pagie and Hogeweg, 2000) we find that even a weak RM system provides a long-term advantage to a bacterial strain when there is a phage that infects both this and other strains of bacteria. Further, we will see that the presence of a RM system becomes necessary when competing bacteria also have an RM system. Further, we argue that a phage with burst size β can facilitate stable coexistence of up to β different bacterial strains, provided that they have unique RM systems—each RM system provides an epigenetic variant of the free phage, and this overcomes the typically strong limits on diversity in mutually supporting phage-bacteria ecosystems (Jover et al., 2013; Haerter et al., 2014).

## 2. Materials and methods

We consider a system of many bacterial strains (*b_i_*, *i* = 1, …, *D*) with different RM systems, all preyed on by a single species of phage (Levin et al., 1988; Frank, 1994). The DNA of each individual phage particle is assumed to carry the methylation obtained in the host from which it emerged, and thus there are also *D* different phage types but they are all simply differently “labeled” versions of the same phage species. We therefore assume that all these different types infect a bacterial strain *b_i_* with the same rate constant η_*i*_ and produce the same lytic burst β*_i_*, both dependent on the bacterial strain only. The imperfection of *b_i_*'s RM system is parametrized by ω_*i*_, the probability that an attacking phage *p_j_*, with a non-protective methylation, completes a lytic cycle and thereby adds β*_i_* phages to the pool of phage *p_i_*. Each bacterial strain also has a specific growth rate γ_*i*_ and a common dilution/death rate α, which together set the maximal total bacterial density, or carrying capacity. This implicitly assumes that all bacteria compete for the same limiting resource. Phage also decay spontaneously at a rate δ_*i*_. The resultant equations are:

(1) $$\frac{db_{i}}{dt}\;=\;\gamma_{i}b_{i}\cdot (1\text{​}-\text{​}B)-\alpha b_{i}-\eta_{i}b_{i}\cdot p_{i}-\eta_{i}\omega_{i}\cdot (P-p_{i})\cdot b_{i},$$

(2) $$\frac{dp_{i}}{dt}\;=\;\beta_{i}\eta_{i}b_{i}\cdot p_{i}+\beta_{i}\eta_{i}\omega_{i}\cdot (P-p_{i})\cdot b_{i}-\eta_{i}B\cdot p_{i}-\delta_{i}p_{i},$$

where *B* = ∑*b_i_* and *P* = ∑*p_i_* are the total bacterial and phage populations, both measured (as are each *b_i_* and *p_i_*) in units of the bacterial carrying capacity at the available food supply rate. Notice that phage species that are completely unmodified could simply be modeled as phage produced by lysis of a bacteria having an RM system with ω_*i*_ = 1.

The figures show dynamics obtained by numerically solving these equations using simple Euler integration implemented in Fortran, but the results have been cross-checked with more accurate integration methods.

## 3. Results

### 3.1. Bacteria having RM systems can invade and out-compete bacteria without RM systems

**Figure 1:**
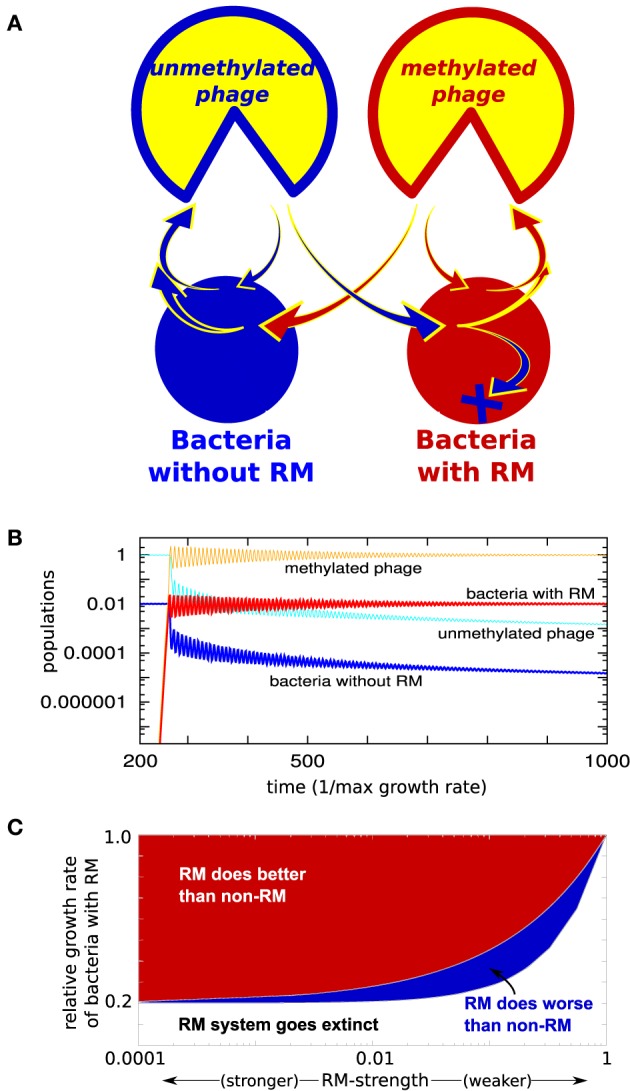
**(A)** Model: Two bacterial strains exposed to one phage type. One of the strains has an RM system. **(B)** Simulation where the “red” strain with weak RM system with ω_1_ = 0.1 is introduced to the “blue” undefended strain (ω_0_ = 1) at time = 250 (measured in units of maximal bacterial growth rate). **(C)** Coexistence of the two bacteria as function of the growth rate of the RM strain, and strength of its RM system, when dilution rate α = 0.2, η = 1, and growth rate of blue strain γ_0_ = 1. As long as growth rate of the RM strain satisfies, γ_1_/γ_0_ < α +(1-α)ω_1_, the RM system will survive. And if $\gamma_{1}/\gamma_{0}>\alpha +(1-\alpha )\sqrt{\omega_{1}}$, then the RM system does better than the non-RM system (see Section 3 of Supplementary Material).

We first investigate a special case, consisting of two bacterial strains: a “red” strain having an RM system (*b*_1_) and a “blue” strain without (*b*_0_), i.e., having ω_0_ = 1 (see Figure 1A). Figure 1B shows what happens when the initial steady state, consisting only of the “blue” bacterial strain and its phage, is invaded by the “red” strain of bacteria, containing a weak RM system. Although the invading strain is introduced at a density 10^−9^ and only has ω_1_ = 0.1, it takes over 99% of the biomass relatively fast. Whenever γ_1_ < γ_0_ (the presence of an RM system should result in slower replication), the non-RM strain is not eliminated completely. However, even if the invading bacteria with an RM system has a substantially lower growth rate, it typically does much better, see Figure 1C. Also note that even a weak RM system has a huge impact on the steady-state biomass of its host—the gain is only proportional to 1−ω_1_, therefore, the steady state difference between, for instance, ω_1_ = 0.1 and ω_1_ = 10^−8^ is only 10%.

It is important to notice that these simple considerations also apply to the case where one bacterial strain has a subset of the RM systems present in the other strain. Thus, our simple considerations imply that even a weak new RM system will allow for invasion of microbial ecosystems.

Our main predictions are that: (i) RM systems provide not only a transient advantage against phage, but also a long-term benefit when competing with bacteria lacking RM systems; (ii) Even weak RM systems are enough to provide this long-term benefit.

These predictions are in agreement with the observation that the majority of bacterial genomes carry at least one RM system (Murray, 2002). Bacteria that do not have any RM systems are typically intracellular pathogens (e.g., Chlamydia, Rickettsia), which are not exposed to phages.

### 3.2. The phage burst size limits the diversity of RM systems

**Figure 2:**
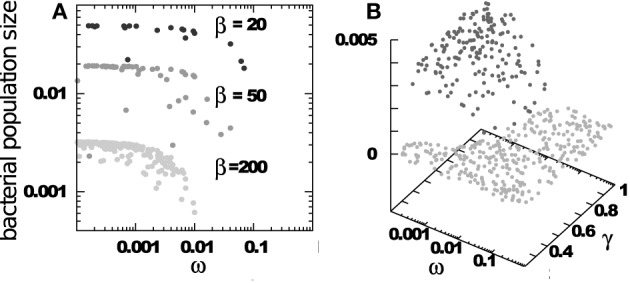
**Final distribution of surviving bacteria when starting with 500 bacterial strains, each having an RM system with strength ω_*i*_ ∈ [0.0001 : 1], growth rate γ_*i*_ ∈ [0.2 : 1]**. Phage adsorption and decay rates are η_*i*_ = 1 and δ_*i*_ = 0.2. The burst sizes vary as indicated. **(A)** Shows bacterial populations vs. RM strengths. **(B)** Shows bacterial populations vs. RM strengths and bacterial growth rates.

The selective pressure on RM systems increases when there are multiple competing bacterial strains. Figure 2 examines a system based on Equations (1) and (2) above, where the parameters β, η (and of course α) do not vary from bacteria to bacteria, but the RM strength ω_*i*_ and the bacterial growth rate γ_*i*_ are different for different bacterial strains. Note that our equations would not change if a bacterial strain has several RM systems provided they are distinct from the RM systems in other strains. Multiple RM systems in one strain are equivalent, in this case, to a single RM system whose effective ω is the product of the individual ωs.

The main predictions from Figure 2 are: (i) The number of surviving bacterial strains, *D*, is about equal to the burst size β of the phage—an intriguing connection between two apparently unrelated numbers; (ii) All surviving strains have ω ≲ 1/β, implying that weak RM systems are under increasing evolutionary pressure in ecosystems with large diversity; (iii) The population size of each bacterial strain is nearly independent of the strength of its RM system, as long as this is stronger that the critical threshold of ≈ 1/β. This “equipartition” of bacterial biomass is also maintained between bacteria with different growth rates, see Figure 2B, reflecting the stabilizing effect of preferential killing of the leading bacterial strain by phages (Thingstad and Lignell, 1997).

Intuitively, these observations are connected: A sustainable coexistence is only possible if, on average, one phage from a burst of β finds a non-immune host cell. Because bacterial biomass is nearly equipartitioned amongst the *D* strains, only a fraction 1/D of phages can reach a non-immune host. Therefore, coexistence requires that β/*D* > 1, i.e., *D* < β.

The *D* ≈ β “rule” can be understood in more detail by considering the simpler case where γ_*i*_ are the same for all bacteria, and only ω_*i*_ varies. Then the total bacterial density of *D* coexisting bacteria is (see Section 2 of Supplementary Material):

(3) $$B=\frac{(\delta /\eta )\sum_{i}\frac{1}{1-\omega_{i}}}{\beta -\sum_{i}\frac{1-\beta \omega_{i}}{\left(1-\omega_{i}\right)}}\approx \frac{\delta }{\beta \eta }\;\cdot \;\frac{D}{1-D/\beta },$$

with the last approximation becoming exact when all the RM systems are sufficiently strong, i.e., ω_*i*_ ≪ 1/β. Here δ/(βη) is the population of one isolated bacterial strain due to predation from the phage (Campbell, 1960), measured in units of the carrying capacity. In order to be consistent, we must have 0 ≤ *B* ≤ γ^*^ (where γ^*^ = (γ − α)/γ). Accordingly, one can only have coexistence of

(4) $$D<\beta \;\cdot \;\frac{\gamma^{*}}{\gamma^{*}+\delta /\eta }$$

distinct bacterial RM systems.

Measured values for phages which infect *E. coli* in good growth conditions give δ/η << 1 (De Paepe and Taddei, 2006), and in this situation we would predict *D*≈β. In oceans with estimated δ = 0.04−0.11 h^−1^ (Noble and Fuhrman, 1997), more phages may die/degrade before reaching a bacteria, and δ/η may become substantial. Then we would predict that less than β strains would be able to coexist. In both cases, β is an upper bound on the number of strains.

### 3.3. Different regimes of selection in an open system

**Figure 3:**
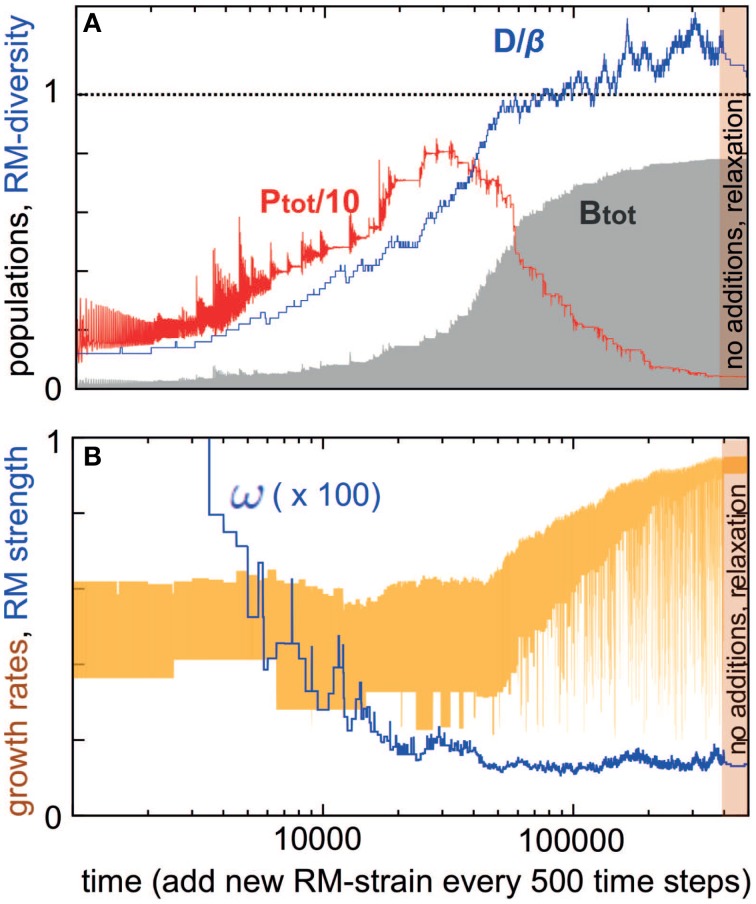
**Development of a system where bacterial strains with new RM systems are added sequentially (500 time steps)**. For the new strain, β*_i_* = 50, η_*i*_ = 1, δ_*i*_ = 0.2, ω_*i*_ is chosen from [0.0001 : 1], and γ*_i_* from [0.2 : 1]. The bacterial dilution rate is fixed at α = 0.2. The simulation starts with few strains, and at the end we let the simulation run for 100000 time units without adding new bacterial strains. **(A)** Shows diversity/burst-size (blue), total bacterial population (gray) and total phage population (red) vs. time. **(B)** Shows RM strengths (blue) and bacterial growth rates (yellow) vs. time.

Above we examined a case where we start with a certain number of RM systems and see how many survive. Such a system would represent a case where multiple strains attempt to invade a new niche, and the predictions from this can be tested in the laboratory using a closed experimental system. Below, we extend this analysis to a situation where new RM systems regularly invade the system. This kind of open system would represent realistic bacterial communities, for example in oceans or soil. Figure 3 shows a simulation starting with *D* = 5 bacterial strains and iterated for 500 time units before introducing a new strain with new ω_*i*_ and γ_*i*_ values. Notice that some of the initial bacteria may be eliminated during these 500 time-units. The simulation subsequently proceeds by adding a new bacterial strain every 500 time-units.

Two “selection regimes” can be identified. First, when *D* is much less than β, the bacterial diversity increases (blue curve in Figure 3A), while at the same time the total bacterial biomass *B* also increases (gray curve in Figure 3A). Bacteria with RM systems gain, both individually and in total, by the presence of more bacteria with different RM systems.

In this regime, the RM systems are under increasing selective pressure, with their average strength increasing (their average ω decreases, as shown by the blue curve in Figure 3B), however, the growth rates of bacteria are *not* under any selection pressure (yellow curve in Figure 3B). Finally, the overall phage abundance also increases with the number of RM systems (red curve in Figure 3A).

The second selection regime appears when the total bacterial density approaches carrying capacity, the diversity *D* becomes close to β, and the weakest of the RM systems has strength <1/β. In this resource-limited regime, the phage population starts declining and bacteria are now selected primarily for high growth rates.

The behavior of the total phage population can again be understood by examining the simple case where only RM strengths vary from bacteria to bacteria. The steady state value of the total phage population then turns out to be related to diversity and bacterial population as follows (see Section 2 of Supplementary Material):

(5) $$P\approx D\;\cdot \;\frac{\gamma (1-B)-\alpha }{\eta }$$

This equation teaches us that *P* first increases with *D*, and then subsequently decreases as *B* approaches the carrying capacity of the available food sources.

This open system with increasing diversity augments the previous main predictions: (i) RM systems provide not just a transient defense against phage, but a long-term benefit both at the level of individual strains and total bacterial biomass; (ii) When there are many bacterial strains with different RM systems, selection favors those which carry RM systems with strength < 1/β (or multiple weaker RM systems with a combined efficiency of < 1/β); (iii) The diversity of coexisting RM systems is limited by the phage burst size; (iv) As diversity approaches its upper limit, the phages die out and the biomass of the bacteria becomes limited by resource, not by phage.

## 4. Discussion

RM systems are intriguing because of their abundance and because they provide easily propagated epigenetic variants of virulent phages. In the absence of such variation, there are strong constraints on the number of phage and bacteria strains that can coexist. For instance, at most two bacterial strains can coexist with a single phage species (Haerter et al., 2014) and even a single bacterial strain might require density-dependent protective mechanisms to enable robust coexistence (Heilmann et al., 2012). As we have shown above, in the presence of RM systems many more bacterial strains (~β) can coexist with a single phage species, even when these strains have quite different growth rates. The RM-derived epigenetic phage variants overcome such constraints because they limit the growth of their specific host. This levels the playing field for the bacterial strains, resulting in an equipartition of the bacterial biomass even amongst strains with very different growth rates. In contrast, other phage defense systems fail to produce as strong a cooperation between bacterial strains, although they may provide stronger individual defenses against phage. For instance, receptor mutants that prevent phage recognition do not act as a sink for phages and therefore do not help other strains. The CRISPR system, which can be thought of as providing “adaptive immunity” as opposed to the “innate immunity” of RM systems (Makarova et al., 2014), also provides a strong defense. Bacteria having it act as a sink for phages, but do not create phage variants at all (Haerter and Sneppen, 2012). Thus, a community of bacterial strains with only CRISPR defenses and one phage predator will not be able to sustain more than two coexisting strains.

The central prediction from our models is that the sustainable diversity of co-existing RM systems is strongly coupled to the phage burst size, as formulated in Equation (4). We find this intriguing because diversity is a population-level parameter, while the burst size is a biophysical parameter that is set by things like the composition and energy content of a single cell. A priori we would not have expected the diversity to be limited so directly by such a biophysical parameter, but our model reveals why RM systems lead to this prediction. Interestingly, the number of reported RM specificities (~300) (Roberts et al., 2010; Seshasayee et al., 2012) is similar to the median burst size of various known phage that infect *E. coli* (De Paepe and Taddei, 2006). Of course, the burst size of a given phage species may vary from host to host and with growth conditions (Weitz and Dushoff, 2008) and is exposed to variation due to occasional mutations. A smaller burst requires a shorter lysis time (Wang, 2006) and should give faster exponential growth at high host density (Abedon et al., 2001). In contrast, abundant diversity of RM systems favors large burst sizes, thus providing an opposing selection pressure. In other words, if a mutant with burst size β_*small*_ < *D* arises in an ecosystem with *D* different RM systems, on average only β_*small*_/*D* < 1 phage from each burst will find a permissive host, so its population will steadily decline until it goes extinct.

Overall, this paper explored the costs and benefits of bacterial RM-systems within the simple framework of mass-action kinetics of a well mixed system. Our investigation was motivated by the expectation, at first glance, that RM systems cannot provide a bacterial strain with a long-term population gain when it is the only host of the phage. Instead, our models show that presence of an RM system allows a bacterial population to use a phage to invade the ecological niche of another bacterial strain that does not have appropriate restriction mechanisms. Furthermore, we suggest RM-systems act as an engine of diversity, helping multiple bacterial strains to co-exist by generating epigenetic variations of the same virulent phage species. Thereby, much of the microbial diversity observed in nature may be supported by this relatively simple DNA recognition and elimination toolbox.

## Author contributions

All authors contributed to conception and design of the study, simulations and analysis, and writing of the manuscript.

### Conflict of interest statement

The authors declare that the research was conducted in the absence of any commercial or financial relationships that could be construed as a potential conflict of interest.
