# Supplementary material for: Restriction modification systems as engines of diversity
Source: Front Microbiol. 2015 Jun 2;6:528. doi: 10.3389/fmicb.2015.00528 (PMC4451750; doi:10.3389/fmicb.2015.00528)
Supplement: Supplementary file 1 [file SupplementaryCalculations.pdf]

Supplementary Material: Restriction Modification Systems as Engines of Diversity

Kim Sneppen [1], Szabolcs Semsey [1], Aswin Seshasayee [2] and Sandeep Krishna [1,2,3,*]

[1] *Center for Models of Life, Niels Bohr Institute, Copenhagen, Denmark*
[2] *National Centre for Biological Sciences, Bangalore, India*
Simons Centre for the Study of Living Machines, National Centre for Biological Sciences, Bangalore, India

Correspondence*:
Sandeep Krishna
National Centre for Biological Sciences, GKVK Campus, Bellary Road, Bangalore 560065, India., sandeep@ncbs.res.in

1 MOST GENERAL MULTI-SPECIES CASE

The most general equations for multiple strains of bacteria, each with a unqiue set of RM systems, in the presence of one species of virulent phage, are those given as equations (1) and (2) in the main text. We repeat these equations below:

$$\frac{db_i}{dt} = \gamma_i b_i(1-B) - \alpha b_i - \eta_i b_i p_i - \eta_i b_i \omega_i (P - p_i), \tag{1}$$

$$\frac{dp_i}{dt} = \beta_i \eta_i b_i p_i + \beta_i \eta_i b_i \omega_i (P - p_i) - \eta_i B p_i - \delta_i p_i, \tag{2}$$

where B and P are the total bacterial and phage populations.

In steady state:

$$\eta_i b_i \left[p_i + \omega_i (P - p_i)\right] = \left[\gamma_i (1-B) - \alpha\right] b_i \tag{3}$$

and

$$\beta_i \eta_i b_i \left[p_i + \omega_i (P - p_i)\right] = \left[\eta_i B + \delta_i\right] p_i. \tag{4}$$

So, firstly, eq. (4) gives

$$\Rightarrow \beta_i \left[\gamma_i (1-B) - \alpha\right] b_i = \left[\eta_i B + \delta_i\right] p_i, \tag{5}$$

$$\Rightarrow \frac{p_i}{b_i} = \frac{\beta_i \left[\gamma_i (1-B) - \alpha\right]}{\left[\eta_i B + \delta_i\right]}. \tag{6}$$

Eq. (3) holds if either $b_i = 0$, or

$$\Rightarrow \eta_i \left[p_i + \omega_i (P - p_i) \right] = \left[\gamma_i (1 - B) - \alpha \right]. \tag{7}$$

Let's assume all $b_i > 0$. Then, from eq. (7), phage individual and total populations satisfy:

$$p_i = \frac{\gamma_i (1 - B) - \alpha - \eta_i \omega_i P}{\eta_i (1 - \omega_i)}, \tag{8}$$

$$\Rightarrow P = \frac{\sum_j \frac{\gamma_j (1-B) - \alpha}{\eta_j (1 - \omega_j)}}{1 + \sum_j \frac{\omega_j}{1 - \omega_j}}. \tag{9}$$

And, from eqs. (6) and (8), bacterial individual and total populations satisfy:

$$b_i = \frac{[\eta_i B + \delta_i]}{\beta_i \left[\gamma_i (1 - B) - \alpha \right]} \times \frac{\gamma_i (1 - B) - \alpha - \eta_i \omega_i P}{\eta_i (1 - \omega_i)}, \tag{10}$$

$$\Rightarrow B = \sum_i \frac{[B + \delta_i / \eta_i]}{\beta_i (1 - \omega_i)} - P \sum_i \frac{[\eta_i B + \delta_i]}{\beta_i \left[\gamma_i (1 - B) - \alpha \right]} \times \frac{\omega_i}{(1 - \omega_i)}. \tag{11}$$

2 SIMPLE CASE WHERE ONLY STRENGTH OF RM SYSTEM VARIES FROM BACTERIA TO BACTERIA

Here, we consider the case where $\gamma, \beta, \eta, \delta$ are all independent of i. Only ω_i can take different values for different i. The simpler equations for this special case seem to capture most of the trends we see in the simulations of the more general case, namely:
(i) RM systems with $\omega < 1/\beta$ are favoured.
(ii) For these, there is approximate "equipartition" of the bacterial biomass amongst the individual bacteria.
(iii) Total bacterial population, B, and the diversity (i.e. number of coexisting bacterial strains), D, are approximately related: $B \propto D/(1 - D/\beta)$, eq. (3) of the main text. This, together with the equipartition, constrains D to be less than the burst size.
(iv) Total phage population, P, increases with diversity when the total bacterial population is small, but decreases with diversity when the total bacterial population approaches the carrying capacity, eq. (5) of the main text.

For this simple case, equations (8) and (9), above, for phage populations, simplify to:

$$\frac{p_i}{b_i} = \frac{\beta \left[\gamma (1 - B) - \alpha \right]}{[\eta B + \delta]} \equiv \lambda, \tag{12}$$

and

$$P = \lambda B = \frac{\beta \left[\gamma (1 - B) - \alpha \right]}{[\eta B + \delta]} B. \tag{13}$$

Using eq. (13), eq. (10) for individual bacterial population then becomes much simpler:

$$b_i = \frac{B}{\beta}\frac{1-\beta\omega_i}{1-\omega_i} + \frac{\delta}{\eta\beta}\frac{1}{1-\omega_i}. \tag{14}$$

From this we can infer a necessary condition for coexistence of all bacteria. For coexistence, $b_i > 0$ for all i,

$$\Rightarrow \omega_i < \frac{1}{\beta}\left(1 + \frac{\delta}{\eta B}\right). \tag{15}$$

This explains why there is a selection pressure on RM system strengths to be approximately less than the inverse of the burst size. Now if $\omega_i \ll 1/\beta$, eq. (14) reduces further to

$$b_i \approx \frac{B}{\beta} + \frac{\delta}{\eta\beta}, \tag{16}$$

that is, there is approximate equipartition of bacterial biomass.

Equation (11) for the total bacterial population simplifies to:

$$\Rightarrow B = \frac{\eta B + \delta}{\beta\eta}\sum_i \frac{1}{(1-\omega_i)} - P\frac{\eta B + \delta}{\beta\left[\gamma(1-B) - \alpha\right]}\sum_i \frac{\omega_i}{(1-\omega_i)}, \tag{17}$$

$$\Rightarrow B = \frac{\eta B + \delta}{\beta\eta}\sum_i \frac{1}{(1-\omega_i)} - \frac{P}{\lambda}\sum_i \frac{\omega_i}{(1-\omega_i)}, \tag{18}$$

$$\Rightarrow B\left[1 - \frac{1}{\beta}\sum_i \frac{1}{(1-\omega_i)} + \sum_i \frac{\omega_i}{(1-\omega_i)}\right] = \frac{\delta}{\beta\eta}\sum_i \frac{1}{1-\omega_i}, \tag{19}$$

$$\Rightarrow B = \frac{(\delta/\eta)\sum_i \frac{1}{1-\omega_i}}{\beta - \sum_i \frac{1-\beta\omega_i}{(1-\omega_i)}}. \tag{20}$$

This is equation (3) in the main text, wherein we explain how this results in the burst size becoming an upper bound on the number of surviving species. Note that, when $\omega_i \ll 1/\beta$, eq. (20) becomes:

$$B \approx \frac{\delta}{\beta\eta}\cdot\frac{D}{1 - D/\beta}, \tag{21}$$

which is also mentioned in eq. (3) of the main text.

Finally, using eq. (21) in eq. (13) gives:

$$P = D\cdot\frac{(\gamma(1-B) - \alpha)}{\eta}, \tag{22}$$

which is equation (5) of the main text.

3 COMPETITION BETWEEN A NON-RM BACTERIAL STRAIN AND ONE WITH AN RM SYSTEM

We investigate the competition between a bacterial strain with no RM system (b_0), i.e., with $\omega_0 = 1$, and a bacterial strain with an RM system (b_1) of strength ω_1, in the presence of one strain of virulent phage that can attack both bacteria. We impose a cost of having the RM system by taking the growth rate of b_1 to be less than the growth rate of the b_0 (i.e. $\gamma_1 \leq \gamma_0$). Time is measured in units of the maximal bacterial growth rate, which is the same as taking $\gamma_0 = 1$. We also assume that η, δ, β and of course α are the same for both bacterial strains. The equations governing the dynamics of this system are a special case of the equations (1) and (2) in the main text and at the beginning of this supplementary material:

$$\frac{db_0}{dt} = b_0(1 - b_0 - b_1) - \alpha b_0 - \eta b_0 p_0 - \eta b_0 p_1 \tag{23}$$

$$\frac{db_1}{dt} = \gamma_1 b_1(1 - b_0 - b_1) - \alpha b_1 - \eta b_1 p_1 - \eta\omega_1 b_1 p_0 \tag{24}$$

$$\frac{dp_0}{dt} = \beta\eta b_0 p_0 + \beta\eta b_0 p_1 - \eta B p_0 - \delta p_0 \tag{25}$$

$$\frac{dp_1}{dt} = \beta\eta b_1 p_1 + \beta\eta\omega_1 b_1 p_0 - \eta B p_1 - \delta p_1 \tag{26}$$

Numerical investigation of the steady-state suggests that:
(i) the RM system goes extinct if $\gamma_1 < \alpha + (1-\alpha)\omega_1$, and
(ii) the RM system does better than the non-RM if $\gamma_1 > \alpha + (1-\alpha)\sqrt{\omega_1}$.

These relations are mentioned in Fig. 1 of the main text, and here we try to derive them analytically. First, in steady-state, from (23) and (24), we get:

$$(1 - b_0 - b_1) = \alpha + \eta p_0 + \eta p_1, \tag{27}$$

$$\gamma_1(1 - b_0 - b_1) = \alpha + \eta p_1 + \eta\omega_1 p_0. \tag{28}$$

Thse can be rewritten as follows:

$$(1 - B) - \alpha = \eta P, \tag{29}$$

$$\gamma_1(1 - B) - \alpha = \eta(1 - \omega_1)p_1 + \eta\omega_1 P, \tag{30}$$

where P and B are the total phage and total bacterial population, respectively.

Now, if the RM system was actually extinct, $b_1 = 0$ and $p_1 = 0$, then, from eqs. (23) and (24), the total phage and bacteria levels would be

$$P = \frac{1}{\eta}\left[1 - \alpha - \frac{\delta}{\eta(\beta - 1)}\right] \tag{31}$$

$$B = \frac{\delta}{\eta(\beta - 1)}. \tag{32}$$

In contrast, if the RM system is close to extinction but not actually extinct, then p_1 is very small compared to P, so eq. (30) can be written:

$$\gamma_1(1-B)-\alpha \approx \eta\omega_1 P, \tag{33}$$

and then combining (29) and (33) gives

$$P=\frac{\alpha(1-\gamma_1)}{\eta(\gamma_1-\omega_1)} \tag{34}$$

$$B=\frac{\gamma_1-\alpha+\alpha\omega_1-\omega_1}{\gamma_1-\omega_1}. \tag{35}$$

For this solution to survive, instead of (31) and (32), a necessary condition is $P \geq 0$ and $B \geq 0$, i.e., we need the following criterion to be satisfied:

$$\gamma_1 \geq \alpha+(1-\alpha)\omega_1, \tag{36}$$

which explains result (i).

An even more accurate formula for the line of extinction can be derived by equating eq. (31) to eq. (34), and (32) to (35). This gives:

$$\left[\frac{(1-\alpha)\,\eta(\beta-1)-\delta}{\eta(\beta-1)}\right]=\frac{\alpha(1-\gamma_1)}{(\gamma_1-\omega_1)} \tag{37}$$

$$\frac{\delta}{\eta(\beta-1)}=\frac{\gamma_1-\alpha+\alpha\omega_1-\omega_1}{\gamma_1-\omega_1}, \tag{38}$$

both of which give the following for the line of extinction:

$$\gamma_1=\frac{\alpha\eta(\beta-1)}{\eta(\beta-1)-\delta}+\omega_1\frac{[(1-\alpha)\,\eta(\beta-1)-\delta]}{\eta(\beta-1)-\delta}. \tag{39}$$

Notice that this reduces to the simpler eq. (36) when β is very large.

Now, we address result (ii). First, from eq. (27) and (28), we get:

$$p_0=\frac{(1-\gamma_1)}{\eta(\gamma_1-\omega_1)}\left(\alpha+\eta p_1\right). \tag{40}$$

Then, from (25) and (26) we get the following, assuming neither bacteria goes extinct:

$$\beta\eta b_0\left(p_0+p_1\right)=\left(\eta B+\delta\right)p_0 \tag{41}$$

$$\beta\eta b_1\left(p_1+\omega_1 p_0\right)=\left(\eta B+\delta\right)p_1. \tag{42}$$

We want to find out when the RM system will do better than the strain without an RM system, so we look at the conditions required to get $b_0=b_1$:

Eq (41) divided by (42) in this case gives:

$$b_0\,(p_0 + p_1)\,p_1 = b_1\,(p_1 + \omega_1 p_0)\,p_0, \tag{43}$$

$$\Rightarrow p_1 = p_0\sqrt{\omega_1}. \tag{44}$$

Putting this into (40):

$$p_0 = \frac{(1-\gamma_1)}{\eta(\gamma_1 - \omega_1)}\left(\alpha + \eta\sqrt{\omega_1}p_0\right), \tag{45}$$

$$\Rightarrow p_0 = \frac{\alpha(1-\gamma_1)/\eta}{\left(\gamma_1 - \sqrt{\omega_1}\right)\left(1+\sqrt{\omega_1}\right)}. \tag{46}$$

Putting (44) and (46) into (27), and again setting $b_0 = b_1$:

$$b_0 = \frac{1}{2}\left[1 - \alpha - \frac{\alpha(1-\gamma_1)}{\left(\gamma_1 - \sqrt{\omega_1}\right)}\right]. \tag{47}$$

Putting (44) and (46) into (41):

$$b_0 = \frac{(\delta/\eta)}{\beta(1+\sqrt{\omega_1}) - 2}. \tag{48}$$

Combining (47) and (48), we get the following criterion for obtaining $b_0 = b_1 > 0$:

$$\gamma_1 = \frac{\eta\left(\sqrt{\omega_1} - \alpha\sqrt{\omega_1} + \alpha\right)\left[\beta(1+\sqrt{\omega_1}) - 2\right] - 2\delta\sqrt{\omega_1}}{\eta\left[\beta(1+\sqrt{\omega_1}) - 2\right] - 2\delta}. \tag{49}$$

Thus, for the RM system strain to do better than the one lacking an RM system its growth rate should be larger than the threshold value given by:

$$\gamma_1 > \frac{\eta\alpha(\beta-2) + \sqrt{\omega_1}\left[\eta(1-\alpha)(\beta-2) + \eta\alpha\beta - 2\delta\right] + \eta\beta(1-\alpha)\omega_1}{\eta(\beta-2) - 2\delta + \eta\beta\sqrt{\omega_1}}. \tag{50}$$

When β is large, this reduces to:

$$\gamma_1 \gtrsim \alpha + (1-\alpha)\sqrt{\omega_1}, \tag{51}$$

which explains result (ii).
